# Supplementary material for: Hot spot prediction in protein-protein interactions by an ensemble system
Source: BMC Syst Biol. 2018 Dec 31;12(Suppl 9):132. doi: 10.1186/s12918-018-0665-8 (PMC6311905; doi:10.1186/s12918-018-0665-8)
Supplement: Supplementary file 2 — All relevant MATLAB code. A simple Matlab implement of our predictor. (ZIP 19,742 kb) [file 12918_2018_665_MOESM2_ESM.zip › Additional file 2/Read ASA/ASA website/SKEMPI/1DQJB.htm]

 NetSurfP - Protein Surface Accessibility and Secondary Structure Predictions

  

|  |  |  |
| --- | --- | --- |
|  |  | NetSurfP - Protein Surface Accessibility and Secondary Structure Predictions  Technical University of Denmark |

  


---

```
# For publication of results, please cite:
# A generic method for assignment of reliability scores applied to solvent accessibility predictions.
# Bent Petersen, Thomas Nordahl Petersen, Pernille Andersen, Morten Nielsen and Claus Lundegaard
# BMC Structural Biology 2009, 9:51 doi:10.1186/1472-6807-9-51
#
# Column 1: Class assignment - B for buried or E for Exposed - Threshold: 25% exposure, but not based on RSA
# Column 2: Amino acid
# Column 3: Sequence name
# Column 4: Amino acid number
# Column 5: Relative Surface Accessibility - RSA
# Column 6: Absolute Surface Accessibility
# Column 7: Z-fit score for RSA prediction
# Column 8: Probability for Alpha-Helix
# Column 9: Probability for Beta-strand
# Column 10: Probability for Coil
E E  1DQJB                  1    0.795 138.817   1.324   0.005   0.015   0.979
B V  1DQJB                  2    0.201  30.955  -0.154   0.003   0.718   0.279
E Q  1DQJB                  3    0.446  79.727   1.437   0.001   0.900   0.099
B L  1DQJB                  4    0.141  25.854   0.861   0.001   0.900   0.099
E Q  1DQJB                  5    0.434  77.530   0.699   0.002   0.816   0.182
B E  1DQJB                  6    0.252  44.024   0.772   0.004   0.514   0.481
E S  1DQJB                  7    0.441  51.697  -0.434   0.004   0.197   0.799
E G  1DQJB                  8    0.511  40.200  -1.866   0.018   0.088   0.893
E P  1DQJB                  9    0.517  73.306  -2.341   0.018   0.047   0.935
E S  1DQJB                 10    0.508  59.538  -1.806   0.020   0.205   0.775
E L  1DQJB                 11    0.399  73.039  -0.732   0.021   0.451   0.528
E V  1DQJB                 12    0.282  43.297   0.624   0.021   0.451   0.528
E K  1DQJB                 13    0.398  81.869   1.246   0.021   0.279   0.699
E P  1DQJB                 14    0.472  66.948  -0.533   0.005   0.045   0.951
E S  1DQJB                 15    0.624  73.121  -1.824   0.005   0.015   0.979
E Q  1DQJB                 16    0.469  83.781  -0.385   0.004   0.138   0.858
E T  1DQJB                 17    0.450  62.360  -0.381   0.004   0.420   0.576
B L  1DQJB                 18    0.147  26.824   0.789   0.002   0.816   0.182
E S  1DQJB                 19    0.434  50.900   0.921   0.001   0.900   0.099
B L  1DQJB                 20    0.081  14.740   0.487   0.001   0.900   0.099
B T  1DQJB                 21    0.206  28.586   0.904   0.001   0.900   0.099
B C  1DQJB                 22    0.045   6.262   0.089   0.001   0.900   0.099
B S  1DQJB                 23    0.308  36.086   0.490   0.001   0.900   0.099
B V  1DQJB                 24    0.126  19.335  -0.557   0.001   0.900   0.099
B T  1DQJB                 25    0.179  24.772  -0.654   0.004   0.616   0.381
B G  1DQJB                 26    0.273  21.485  -1.827   0.004   0.197   0.799
B D  1DQJB                 27    0.256  36.962  -1.162   0.005   0.336   0.660
B S  1DQJB                 28    0.191  22.432  -0.976   0.004   0.514   0.481
B V  1DQJB                 29    0.116  17.814  -0.941   0.004   0.420   0.576
B T  1DQJB                 30    0.248  34.398  -1.251   0.021   0.279   0.699
B S  1DQJB                 31    0.269  31.574  -1.293   0.018   0.088   0.893
B D  1DQJB                 32    0.137  19.684   0.162   0.018   0.088   0.893
B Y  1DQJB                 33    0.097  20.665   0.019   0.022   0.359   0.619
B W  1DQJB                 34    0.031   7.431   0.341   0.021   0.756   0.223
B S  1DQJB                 35    0.050   5.860   0.107   0.018   0.846   0.136
B W  1DQJB                 36    0.037   8.802   0.343   0.018   0.846   0.136
B I  1DQJB                 37    0.039   7.252   0.148   0.018   0.846   0.136
B R  1DQJB                 38    0.127  29.014   0.477   0.021   0.756   0.223
B K  1DQJB                 39    0.092  18.904   0.510   0.021   0.451   0.528
E F  1DQJB                 40    0.265  53.206   0.837   0.005   0.045   0.951
E P  1DQJB                 41    0.389  55.242   0.711   0.005   0.015   0.979
E G  1DQJB                 42    0.621  48.912  -1.962   0.005   0.015   0.979
E N  1DQJB                 43    0.434  63.523   0.441   0.018   0.019   0.964
E K  1DQJB                 44    0.473  97.317  -0.507   0.018   0.088   0.893
B L  1DQJB                 45    0.179  32.738   0.917   0.005   0.336   0.660
B E  1DQJB                 46    0.103  18.081  -0.286   0.003   0.718   0.279
B Y  1DQJB                 47    0.054  11.583   0.337   0.001   0.900   0.099
B M  1DQJB                 48    0.024   4.862   0.886   0.001   0.959   0.040
B G  1DQJB                 49    0.019   1.480  -0.168   0.001   0.959   0.040
B Y  1DQJB                 50    0.099  21.156  -0.074   0.001   0.959   0.040
B I  1DQJB                 51    0.034   6.346  -0.012   0.001   0.959   0.040
B S  1DQJB                 52    0.193  22.573   0.541   0.003   0.718   0.279
E Y  1DQJB                 53    0.358  76.505  -0.763   0.004   0.197   0.799
E S  1DQJB                 54    0.615  72.101  -2.292   0.005   0.015   0.979
E G  1DQJB                 55    0.475  37.375  -2.118   0.016   0.005   0.979
E S  1DQJB                 56    0.522  61.167  -0.797   0.018   0.047   0.935
E T  1DQJB                 57    0.353  48.919  -0.283   0.019   0.141   0.840
E Y  1DQJB                 58    0.451  96.315   0.092   0.020   0.205   0.775
B Y  1DQJB                 59    0.226  48.211   0.557   0.056   0.142   0.802
B H  1DQJB                 60    0.280  50.932  -0.338   0.053   0.043   0.903
E P  1DQJB                 61    0.673  95.513  -0.622   0.018   0.047   0.935
E S  1DQJB                 62    0.457  53.537  -0.024   0.018   0.047   0.935
B L  1DQJB                 63    0.101  18.456  -0.532   0.019   0.141   0.840
E K  1DQJB                 64    0.450  92.606   0.875   0.019   0.141   0.840
B S  1DQJB                 65    0.262  30.730  -1.559   0.005   0.262   0.733
B R  1DQJB                 66    0.229  52.395   0.575   0.003   0.718   0.279
B I  1DQJB                 67    0.060  11.119  -0.529   0.001   0.900   0.099
B S  1DQJB                 68    0.152  17.850   0.276   0.001   0.959   0.040
B I  1DQJB                 69    0.058  10.693   0.595   0.001   0.959   0.040
B T  1DQJB                 70    0.117  16.283   0.063   0.003   0.718   0.279
E R  1DQJB                 71    0.293  67.189   0.515   0.022   0.552   0.426
B D  1DQJB                 72    0.274  39.483  -0.656   0.005   0.336   0.660
E T  1DQJB                 73    0.480  66.521  -0.325   0.018   0.088   0.893
E S  1DQJB                 74    0.292  34.222  -1.754   0.018   0.047   0.935
E K  1DQJB                 75    0.519 106.697   0.704   0.019   0.141   0.840
E N  1DQJB                 76    0.261  38.269  -0.328   0.005   0.336   0.660
B Q  1DQJB                 77    0.221  39.506   1.182   0.002   0.816   0.182
B Y  1DQJB                 78    0.079  16.925  -0.579   0.006   0.962   0.032
B Y  1DQJB                 79    0.174  37.184   0.364   0.001   0.959   0.040
B L  1DQJB                 80    0.049   9.009   0.550   0.001   0.959   0.040
B Q  1DQJB                 81    0.199  35.506  -0.021   0.001   0.900   0.099
B L  1DQJB                 82    0.088  16.204  -0.214   0.003   0.718   0.279
E N  1DQJB                 83    0.396  57.916  -0.037   0.005   0.336   0.660
E S  1DQJB                 84    0.418  48.978  -0.858   0.004   0.197   0.799
B V  1DQJB                 85    0.146  22.502   0.166   0.018   0.088   0.893
E T  1DQJB                 86    0.488  67.741   0.907   0.018   0.047   0.935
E T  1DQJB                 87    0.390  54.051  -0.628   0.052   0.084   0.864
E E  1DQJB                 88    0.416  72.675  -0.670   0.052   0.084   0.864
B D  1DQJB                 89    0.184  26.500  -0.848   0.052   0.084   0.864
B T  1DQJB                 90    0.130  18.045   0.896   0.066   0.296   0.638
B A  1DQJB                 91    0.038   4.210  -0.317   0.085   0.775   0.140
B T  1DQJB                 92    0.074  10.278   0.677   0.085   0.775   0.140
B Y  1DQJB                 93    0.038   8.056   0.759   0.085   0.775   0.140
B Y  1DQJB                 94    0.063  13.506   0.939   0.085   0.775   0.140
B C  1DQJB                 95    0.022   3.061   1.111   0.087   0.683   0.230
B A  1DQJB                 96    0.040   4.430  -0.044   0.074   0.484   0.442
B S  1DQJB                 97    0.180  21.108  -0.174   0.066   0.296   0.638
E W  1DQJB                 98    0.359  86.339  -0.868   0.052   0.084   0.864
B G  1DQJB                 99    0.269  21.139  -0.836   0.053   0.043   0.903
B G  1DQJB                100    0.183  14.402  -1.160   0.052   0.084   0.864
B D  1DQJB                101    0.201  29.007  -0.823   0.056   0.142   0.802
B V  1DQJB                102    0.107  16.446  -0.453   0.064   0.216   0.721
B W  1DQJB                103    0.066  15.825  -0.476   0.064   0.216   0.721
B G  1DQJB                104    0.039   3.101  -3.976   0.020   0.205   0.775
B A  1DQJB                105    0.147  16.232  -0.283   0.019   0.141   0.840
B G  1DQJB                106    0.068   5.383  -1.811   0.018   0.088   0.893
B T  1DQJB                107    0.220  30.583  -0.159   0.018   0.088   0.893
E T  1DQJB                108    0.291  40.431  -0.380   0.020   0.205   0.775
B V  1DQJB                109    0.042   6.502  -1.188   0.022   0.359   0.619
B T  1DQJB                110    0.124  17.199  -2.316   0.022   0.359   0.619
B V  1DQJB                111    0.159  24.423  -1.041   0.022   0.359   0.619
B S  1DQJB                112    0.255  29.921  -0.966   0.021   0.279   0.699
E S  1DQJB                113    0.381  44.618  -1.716   0.018   0.088   0.893
E A  1DQJB                114    0.484  53.282  -1.146   0.053   0.043   0.903
E K  1DQJB                115    0.503 103.488  -1.155   0.053   0.043   0.903
E T  1DQJB                116    0.437  60.543  -1.004   0.053   0.043   0.903
E T  1DQJB                117    0.406  56.340  -1.293   0.053   0.043   0.903
E A  1DQJB                118    0.399  44.014  -1.494   0.018   0.019   0.964
B P  1DQJB                119    0.330  46.813  -2.013   0.018   0.047   0.935
B S  1DQJB                120    0.287  33.590  -1.676   0.019   0.141   0.840
B V  1DQJB                121    0.282  43.390  -1.394   0.020   0.205   0.775
B Y  1DQJB                122    0.238  50.946  -2.349   0.021   0.279   0.699
E P  1DQJB                123    0.336  47.721  -1.679   0.021   0.279   0.699
B L  1DQJB                124    0.243  44.402  -2.059   0.020   0.205   0.775
E A  1DQJB                125    0.366  40.300  -1.686   0.018   0.088   0.893
E P  1DQJB                126    0.567  80.443  -2.263   0.018   0.047   0.935
E V  1DQJB                127    0.410  62.956  -2.513   0.005   0.045   0.951
B C  1DQJB                128    0.294  41.292  -2.197   0.005   0.015   0.979
E G  1DQJB                129    0.623  49.062  -2.378   0.005   0.015   0.979
E D  1DQJB                130    0.566  81.546  -2.629   0.018   0.019   0.964
E T  1DQJB                131    0.450  62.373  -1.451   0.018   0.019   0.964
E T  1DQJB                132    0.455  63.150  -0.812   0.018   0.019   0.964
E G  1DQJB                133    0.492  38.697  -1.396   0.018   0.047   0.935
E S  1DQJB                134    0.558  65.421  -1.550   0.004   0.085   0.910
E S  1DQJB                135    0.398  46.587   0.522   0.005   0.336   0.660
B V  1DQJB                136    0.075  11.604   0.507   0.002   0.816   0.182
B T  1DQJB                137    0.229  31.707   0.781   0.001   0.900   0.099
B L  1DQJB                138    0.037   6.848  -0.415   0.001   0.959   0.040
B G  1DQJB                139    0.056   4.376  -0.041   0.001   0.900   0.099
B C  1DQJB                140    0.029   4.128   0.019   0.001   0.900   0.099
B L  1DQJB                141    0.153  27.996   0.285   0.001   0.900   0.099
B V  1DQJB                142    0.043   6.532  -0.601   0.003   0.718   0.279
B K  1DQJB                143    0.285  58.563  -0.365   0.005   0.336   0.660
E G  1DQJB                144    0.465  36.588  -2.120   0.004   0.138   0.858
B Y  1DQJB                145    0.207  44.279  -0.861   0.004   0.197   0.799
E F  1DQJB                146    0.328  65.789  -0.498   0.004   0.197   0.799
B P  1DQJB                147    0.246  34.893  -1.517   0.004   0.197   0.799
E E  1DQJB                148    0.575 100.383  -1.044   0.004   0.138   0.858
E P  1DQJB                149    0.446  63.273  -1.762   0.004   0.197   0.799
B V  1DQJB                150    0.205  31.585   0.002   0.003   0.718   0.279
B T  1DQJB                151    0.258  35.785  -0.488   0.001   0.900   0.099
B L  1DQJB                152    0.051   9.356   0.063   0.001   0.959   0.040
B T  1DQJB                153    0.195  27.102   0.063   0.011   0.918   0.071
B W  1DQJB                154    0.062  14.935   0.222   0.011   0.918   0.071
B N  1DQJB                155    0.237  34.653   0.099   0.002   0.816   0.182
E S  1DQJB                156    0.407  47.724  -0.586   0.004   0.616   0.381
E G  1DQJB                157    0.394  30.992  -0.351   0.004   0.138   0.858
E S  1DQJB                158    0.532  62.386  -0.013   0.005   0.015   0.979
E L  1DQJB                159    0.562 102.939  -0.475   0.005   0.015   0.979
E S  1DQJB                160    0.663  77.668  -1.901   0.005   0.015   0.979
E S  1DQJB                161    0.536  62.866   0.207   0.005   0.045   0.951
E G  1DQJB                162    0.457  35.950  -0.407   0.004   0.138   0.858
B V  1DQJB                163    0.174  26.744  -0.414   0.004   0.514   0.481
E H  1DQJB                164    0.343  62.301   0.132   0.001   0.900   0.099
B T  1DQJB                165    0.243  33.676  -0.531   0.001   0.900   0.099
B F  1DQJB                166    0.184  36.949   1.513   0.002   0.816   0.182
B P  1DQJB                167    0.102  14.531  -0.637   0.004   0.616   0.381
B A  1DQJB                168    0.234  25.787  -1.110   0.002   0.816   0.182
B V  1DQJB                169    0.185  28.434  -1.001   0.001   0.900   0.099
E L  1DQJB                170    0.302  55.278  -0.008   0.003   0.718   0.279
E Q  1DQJB                171    0.561 100.230  -1.310   0.005   0.336   0.660
E S  1DQJB                172    0.732  85.837  -1.893   0.005   0.015   0.979
E D  1DQJB                173    0.576  83.002  -2.607   0.005   0.045   0.951
E L  1DQJB                174    0.430  78.751  -0.016   0.004   0.420   0.576
B Y  1DQJB                175    0.254  54.301  -1.377   0.004   0.616   0.381
B T  1DQJB                176    0.206  28.628  -0.783   0.002   0.816   0.182
B L  1DQJB                177    0.141  25.854  -0.877   0.001   0.900   0.099
B S  1DQJB                178    0.246  28.808  -0.077   0.001   0.959   0.040
B S  1DQJB                179    0.034   4.043  -0.842   0.001   0.959   0.040
B S  1DQJB                180    0.183  21.483   0.353   0.001   0.959   0.040
B V  1DQJB                181    0.038   5.764  -0.163   0.001   0.959   0.040
B T  1DQJB                182    0.190  26.284   0.915   0.001   0.959   0.040
B V  1DQJB                183    0.049   7.577   1.104   0.001   0.900   0.099
E T  1DQJB                184    0.304  42.095   1.108   0.004   0.616   0.381
E S  1DQJB                185    0.329  38.571  -0.381   0.021   0.279   0.699
E S  1DQJB                186    0.604  70.777  -0.874   0.005   0.336   0.660
E T  1DQJB                187    0.538  74.593   0.393   0.004   0.197   0.799
B W  1DQJB                188    0.214  51.467  -0.977   0.004   0.085   0.910
E P  1DQJB                189    0.723 102.608   1.039   0.005   0.045   0.951
E S  1DQJB                190    0.733  85.943   0.996   0.005   0.015   0.979
E Q  1DQJB                191    0.501  89.550   0.971   0.004   0.420   0.576
E S  1DQJB                192    0.424  49.716   1.254   0.001   0.900   0.099
B I  1DQJB                193    0.077  14.171   0.825   0.001   0.959   0.040
E T  1DQJB                194    0.323  44.842   1.799   0.001   0.959   0.040
B C  1DQJB                195    0.029   4.015   0.135   0.001   0.959   0.040
E N  1DQJB                196    0.354  51.870   1.056   0.001   0.959   0.040
B V  1DQJB                197    0.098  15.124   0.033   0.001   0.900   0.099
E A  1DQJB                198    0.550  60.610   0.533   0.001   0.900   0.099
B H  1DQJB                199    0.196  35.689   0.473   0.004   0.514   0.481
E P  1DQJB                200    0.678  96.251   0.404   0.005   0.045   0.951
E A  1DQJB                201    0.717  79.046  -1.271   0.005   0.015   0.979
E S  1DQJB                202    0.547  64.062  -0.526   0.005   0.045   0.951
E S  1DQJB                203    0.572  67.027  -0.125   0.004   0.138   0.858
E T  1DQJB                204    0.448  62.193   0.852   0.004   0.420   0.576
E K  1DQJB                205    0.508 104.496   1.382   0.003   0.718   0.279
B V  1DQJB                206    0.143  21.964   0.151   0.001   0.900   0.099
E D  1DQJB                207    0.564  81.287   0.497   0.001   0.900   0.099
E K  1DQJB                208    0.371  76.253   0.648   0.001   0.900   0.099
E K  1DQJB                209    0.572 117.722   1.058   0.003   0.718   0.279
E I  1DQJB                210    0.529  97.920  -0.684   0.003   0.003   0.994

Explain the output.  Go back.
```

---
